# Development and evaluation of a rapid and sensitive multienzyme isothermal rapid amplification with a lateral flow dipstick assay for detection of *Acinetobacter baumannii* in spiked blood specimens

**DOI:** 10.3389/fcimb.2022.1010201

**Published:** 2022-10-19

**Authors:** Wei-Wei Hu, Jian-Wei He, Shu-Liang Guo, Jin Li

**Affiliations:** ^1^ Department of Respiratory and Critical Care Medicine, The First Affiliated Hospital of Chongqing Medical University, Chongqing, China; ^2^ Key Laboratory of Clinical Laboratory Diagnostics (Ministry of Education), College of Laboratory Medicine, Chongqing Medical University, Chongqing, China

**Keywords:** multienzyme isothermal rapid amplification, lateral flow dipstick, *Acinetobacter baumannii*, bloodstream infection, spiked blood specimens

## Abstract

**Purpose:**

This study aimed to establish the multienzyme isothermal rapid amplification with a lateral flow dipstick (MIRA-LFD) assay and evaluate its performance in detection of *A. baumannii* in spiked blood specimens.

**Methods:**

The study was divided into two stages: a pilot study to establish the methodology and a clinical validation study to evaluate its performance. In the first step, we designed primers specific to detect *A. baumannii*, optimized the MIRA-LFD assay and analyzed its performance regarding limits of detection, reproducibility, specificity, and efficiency of detection using real-time PCR method. In the second step, we obtained 50 spiked blood isolates and detected these pathogens by MIRA-LFD assay. The MIRA-LFD time was 15 min from DNA sample amplification to complete pathogen detection.

**Results:**

The developed MIRA-LFD assay displayed a detection limit of 6 CFU/mL for detecting *A. baumannii*, which was significantly better than that of real-time PCR method, and no cross-reactivity was observed in other non-*A. baumannii* studied. The results obtained with 50 spiked blood isolates suggested that the developed MIRA-LFD assay had high specificity and sensitivity for identifying *A. baumannii*.

**Conclusions:**

This study demonstrates that the established MIRA-LFD assay is time-saving, more effective and sensitive, which may become a powerful tool for rapid and reliable diagnosis of bloodstream infection caused by *A. baumannii* in primary hospitals.

## Introduction

*Acinetobacter baumannii* is an opportunistic pathogen, which is widely distributed in nature and can survive in hospital environment for a long time (Zarrilli et al., 2009). It has the characteristics of strong survivability and resistance, high colonization rate and bacterial resistance rate (Perez et al., 2007). *A. baumannii* is the main pathogen causing nosocomial infection and bloodstream infection (BSI) (Chopra et al., 2013). Compared with other pathogens, *A. baumannii* has a more serious drug resistance situation (Kengkla et al., 2018; Tada et al., 2020; Liu et al., 2021). Furthermore, the resistance rate of *A. baumannii* to commonly used antibiotics was more than 70% (Hu et al., 2019). Therefore, a rapid and accurate diagnostic assay is required for the effective control the BSI caused by *A. baumannii.*

To date, several approaches have been proposed for detecting *A. baumannii*. The most sensitive and applicable for strain discrimination are the well-known molecular detection method (Schaad and Schuenzel, 2010). At first, a PCR assay have been developed for *A. baumannii* based on the *OXA-51* gene (Turton et al., 2006; Abhari et al., 2021). While, the PCR assay for identification of *A. baumannii* is time-consuming and the result is not easy to read, as well as require complex thermal cycling instrument, which are not suitable for low-resource areas. Meanwhile, the isothermal amplification technology has been gradually applied to bacterial detection (Li and Macdonald, 2015; Lobato and O’sullivan, 2018). It has much lower requirements for amplification instruments and faster reaction kinetics. The most representative technologies are RPA (recombinase polymerase amplification) and MIRA (multienzyme isothermal rapid amplification) (Piepenburg et al., 2006; Lu et al., 2021). They can amplify complex DNA targets at room temperature within 10 min, which is more advantageous than other isothermal amplification methods (Shang et al., 2021; Sun et al., 2021; Tu et al., 2022). In the detection format of MIRA amplifier, lateral flow dipstick (LFD) based MIRA detection has been widely established for various detection target (Li et al., 2019a; Li et al., 2021). In order to meet the needs of rapid detection of first aid and emergency treatment, especially for laboratories with limited resources and poor equipment, MIRA-LFD assay is an ideal choice. The aim of the present study was to establish the MIRA-LFD assay and evaluate its performance in detection of *A. baumannii* in spiked blood specimens.

## Materials and methods

### Bacterial strains and DNA preparation

The fifty spiked blood isolates used in verification assays were as follows: thirty *A. baumannii*, four *E. coli*, four *K. pneumoniae*, four *E. cloacae*, two *K. oxytoca*, two *P. aeruginosa*, two *S. maltophilia*, and two *B. cepacia* isolates. For the calculation of bacterial colony forming unit (CFU), the main method was as follows: the bacterial solution with 0.5 McFarland was continuously diluted 10 times, and 10 μL of the diluent (10^6^, 10^7^, 10^8^) with appropriate concentration was dropped on the LB plate. Each concentration was repeated three times, cultured at 37°C for 48 h, and CFU was counted. For spiked blood specimens, 900 μL blood samples were added with 100 μL different concentrations of bacterial strains. The spiked blood specimens were firstly lysed using Red Blood Cell Lysis Buffer (Sansure Biotech Inc., Hunan, China) before bacterial DNA was extracted by TIANamp Bacteria DNA Kit (Tiangen Biotech Co., Ltd., Beijing, China) according to the manufacturer’s instructions. The blood samples used in the experiment were collected from 10 healthy individuals. The extracted DNA was stored at -20°C until next use.

### Identification of isolates by MALDI-TOF MS

The identification of all isolates used in this study was confirmed at the species level by MALDI-TOF MS (bioMerieux, France). According to the manufacturer’s instructions, the mass spectrometry identification results were matched at the highest level by comparing with the *In Vitro* Diagnosis (IVD) database.

### Primers and probes of MIRA-LFD assay

After a systematic literature search and sequence alignment with DNAMAN software, *OXA51* gene of *A. baumannii* was identified. Primer sets and corresponding nfo probes were designed. All primers and probes of MIRA assays were synthesized and purified by BGI Tech Solutions Beijing Liuhe Co., Lid using polyacrylamide gel electrophoresis (PAGE) and high-performance liquid chromatography (HPLC) respectively. The sequences of primers and probes were showed in **Table 1**.

**Table 1:** Primers and probe used in the present study.

Assay	Name	Sequence (5´-3´) and modification	Length (bp)
Basic MIRA	Aba-F1	CAACCACCACAGAAGTATTTAAGTGGGACGGGC	33
	Aba-F2	CTATTCCCAGAATGGGAAAAGGACATGACC	30
	Aba-F3	TAGGCGATGCTATGAAAGCTTCCGCTATTCCG	32
	Aba-F4	TTTATCAAGATTTAGCTCGTCGTATTGGAC	30
	Aba-R1	TGTAAGCAAACTGTGCCTCTTGCTGAGGAG	31
	Aba-R2	TTGGGCTAAATGGAAGCGTTTTATTAGCTAG	31
	Aba-R3	TGAATAACATGGATTGCACTTCATCTTGGAC	31
	Aba-R4	CAGTTAACCAGCCTACTTGTGGGTCTACATC	31
LF-MIRA	Aba-F1	CAACCACCACAGAAGTATTTAAGTGGGACGGGC	33
	Aba-R2	[biotin]CGGCTGGGCCTGGGCCATGACCACGCTGAC	31
	nfo-Aba	[FAM]CCGGTAACCAGCTCAGCCACATGTCGCCGATC[THF]ACACCATCGAGATGG[C3spacer]	47
Real-time PCR	OXA-51-F	TTTAGCTCGTCGTATTGGACT	21
	OXA-51-R	CCTCTTGCTGAGGAGTAATTTT	22
	OXA-51-P	Cy5-TGGCAATGCAGATATCGGTACCCA-BHQ1	24

F, forward primer; R, reverse primer; P, probe; THF, tetrahydrofuran.

### Basic MIRA and MIRA-LFD reactions

A series of Basic MIRA reactions were achieved to screen out the best efficiency primers for subsequent MIRA-LFD reactions. For Basic MIRA kits (Amp-Future Biotech Co., Ltd., Weifang, China), the reaction contained 29.4 μL A buffer, 12.1 μL double-distilled water, 2 μL forward primer (10 μM), 2 μL reverse primer (10 μM), 2 μL sample and 2.5 µL B buffer (280 mM). The mixture was place in a metal heat block at 40°C for 20 min. Finally, the Basic MIRA products were purified by phenol-chloroform method (Solarbio, Beijing, China) and analyzed by 1.5% agarose gel electrophoresis. A total of 16 pairs of specific primers were tested by of basic MIRA, and the best efficiency primers were chosen for MIRA-LFD. A FAM-labeled probe was designed for MIRA-LFD according to the description of MIRA nfo kit (Amp-Future Biotech Co., Ltd., Weifang, China). For MIRA nfo kits (Amp-Future Biotech Co., Ltd., Weifang, China), each reaction included 29.4 μL A buffer, 8.5 μL double-distilled water, 2 μL forward primer (10 μM), 2 μL reverse primer (10 μM), 0.6 μL nfo probe (10 μM), 5 μL sample and 2.5 µL B buffer (280 mM). As **Figure S1** shows, the MIRA reactions were place in a metal bath at 40°C for 10 min. Thereafter, the MIRA products were detected by lateral flow dipsticks (Amp-Future Biotech Co., Ltd., Weifang, China). The amplicons of MIRA were diluted 20-fold in buffer (Milenia Biotec GmbH, Germany). Then lateral flow dipsticks were placed vertically in tubes containing the diluted MIRA products for 5 min.

### Optimization of temperature and time for MIRA-LFD assay

The optimal amplification reaction temperature and time were determined by various temperature settings ranging from 20 to 50°C and examining different time (0-35 min). The MIRA-LFD assay was performed using 3 ng genomic DNA of *A. baumannii* (ATCC19606). In this study, we selected double-distilled water as the negative control. The experiment was carried out in a single reaction and repeated independently three times.

### Specificity and sensitivity of MIRA-LFD assay

To verify the specificity of MIRA-LFD assay, 3 ng genomic DNA of *E. coli*, *K. pneumoniae*, *E. cloacae*, *K. oxytoca*, *P. aeruginosa*, *S. maltophilia*, and *B. cepacia* from spiked blood specimens was examined to identify possible cross-reactions. To evaluate the sensitivity of MIRA-LFD assay from spiked blood specimens, 10-fold serial dilutions of *A. baumannii* ATCC19606, ranging from 6×10^5^ CFU/mL to 6×10^0^ CFU/mL per reaction. The MIRA-LFD assay was prepared according to above MIRA-LFD conditions. The experiment was repeated three times with the same result.

### Real-time PCR assay

To compare their sensitivities, the diluted DNA samples of *A. baumannii* ATCC19606 (6×10^5^ CFU/mL - 6×10^0^ CFU/mL) were tested in parallel by an established real-time PCR protocol (Hamouda, 2017). The reaction included 2.0 µL of DNA template, 12.5 µL of Premix Ex Taq (Probe qPCR) (2X), 8.5 µL of double-distilled water, 0.5 µL of forward primer (10 µM), 0.5 µL of reverse primer (10 µM), and 1.0 µL of the probe (10 µM). The reaction was performed on CFX96 real-time PCR detection system (Bio-Rad, USA). A threshold cycle (Ct value) < 38 was determined as the positive sample. Distilled water was used as negative control. A similar experiment was carried out three times with the same result.

### Evaluation of spiked blood specimens with the MIRA-LFD assay

The feasibility of MIRA-LFD assay used for detecting *A. baumannii* in spiked blood specimens was further investigated. In order to perform the second step experiments, 50 spiked blood isolates were collected to simulate spiked blood specimens (6×10^4^ CFU/mL). DNA extraction of such specimens and MIRA-LFD assay were conducted as described above. The performance of the newly developed MIRA-LFD assay was compared to that of MALDI-TOF MS. The experiment was repeated three times with the same result.

## Results

### Primer screening and identification

MIRA is a multienzyme-assisted isothermal amplification technique (Ma et al., 2016; Xiong et al., 2020). As primers playing an important role in the amplification process, the combination of different primers will have various amplification effects. Therefore, a series of primer screening experiments before MIRA-LFD are necessary. According to manufacturer’s instructions, we screened 16 pairs of forward and reverse primers by using basic MIRA reaction. As shown in **Figure S2**, the best primer set for *A. baumannii* was identified as R2/F1 based on the recommendations for MIRA and the brightness of the electrophoretic bands. Finally, the primer and probe sequences for MIRA-LFD were determined according to the selected primer sets.

### Optimization of the reaction temperature and time

**Figure 1:**
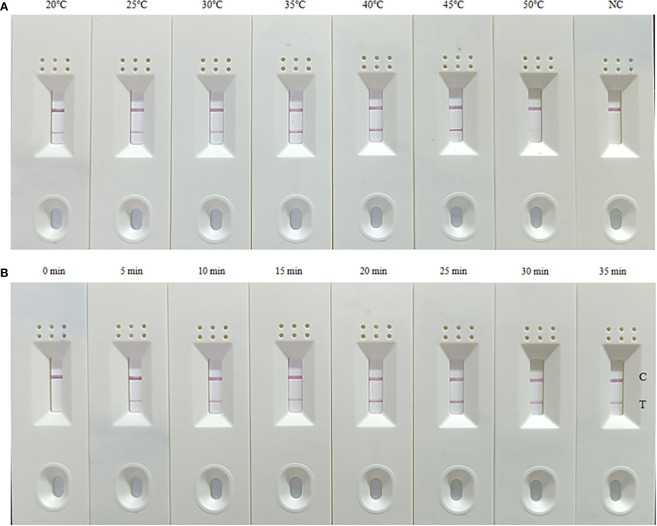
Optimization of the temperature **(A)** and time **(B)** for the MIRA-LFD assay. **(A)** The optimal amplification reaction time was determined by examining various temperature settings ranging from 20 to 50°C; **(B)** The optimal amplification temperature was determined by examining different time (0-35 min). NC, negative control; C, control line; T, test line. These experiments were repeated three times.

In order to determine the optimal amplification temperature, the MIRA-LFD assay was performed according to the manufacturers. The best results are obtained between 25°C and 45°C, and the test lines on the side lateral flow dipstick can be observed in a wide temperature range (**Figure 1A**). To assess the shortest amplification time, the test band can be observed during the amplification time of 5 min (**Figure 1B**). Taking into account the detection efficiency and sensitivity, the amplification time might be sufficient for 10 min. Therefore, the whole test including MIRA amplification and lateral flow dipsticks detection is just 15 min or less.

### Specificity determination of MIRA-LFD assay

**Figure 2:**
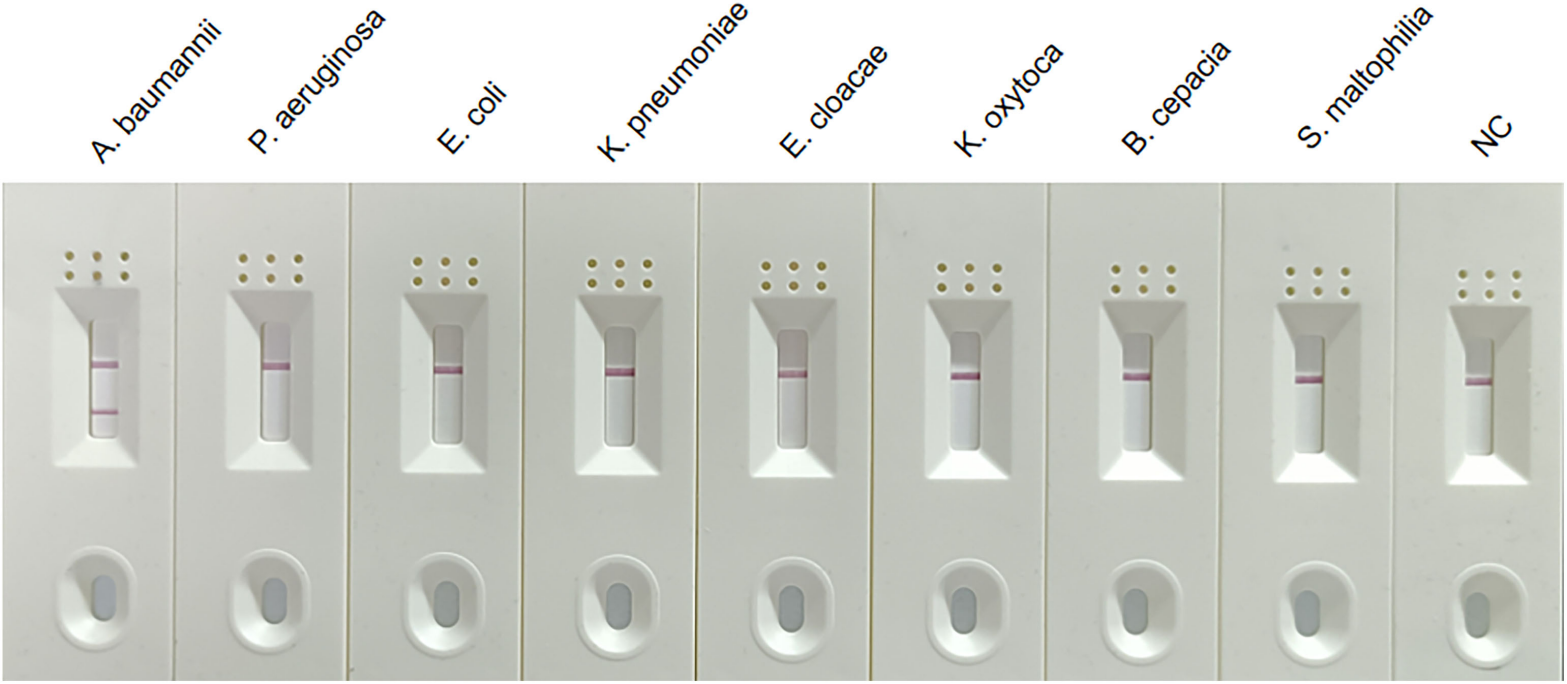
Specificity of the MIRA-LFD assay. Results showed that only the positive control sample and *A. baumannii* isolates produced amplification signals, whereas other non-*A. baumannii* isolates and negative control produced no amplification signals. NC, negative control; C, control line; T, test line.

The specificity of the MIRA-LFD assay for *A. baumannii* was confirmed by detecting *E. coli*, *K. pneumoniae*, *E. cloacae*, *K. oxytoca*, *P. aeruginosa*, *S. maltophilia*, and *B. cepacia*. As can be seen in **Figure 2**, only *A. baumannii* showed both control and test lines, while no test lines were obtained from the other dipsticks, demonstrating that established MIRA-LFD assay have good specificity and no cross-reactions occurred.

### Sensitivity determination of MIRA-LFD assay

**Figure 3:**
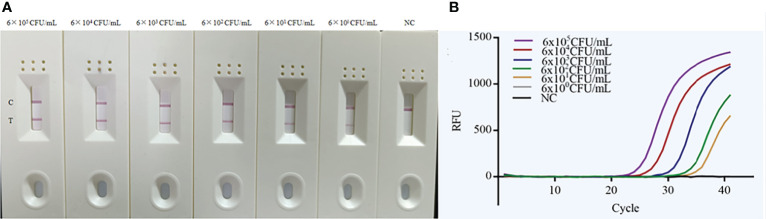
Sensitivity of the MIRA-LFD assay and the real-time PCR assay. **(A)** Serially diluted gDNA of targeted bacteria (6×10^5^ CFU/mL, 6×10^4^ CFU/mL, 6×10^3^ CFU/mL, 6×10^2^ CFU/mL, 6×10^1^ CFU/mL and 6×10^0^ CFU/mL per reaction) was tested by MIRA-LFD at 40°C for 10 min. This experiment was repeated four times for low-concentrated samples (6 CFU/mL). NC, negative control; C, control line; T, test line. **(B)** The quantity of genomic DNA of *A baumannii* was tested by real-time PCR at 95°C for 5 min, followed by 40 cycles of 95°C for 10 seconds and 60°C for 30 seconds.

The sensitivity of the MIRA-LFD assay was determined using a concentration of 6×10^5^ CFU/mL to 6×10^0^ CFU/mL of bacterial DNA extracted from spiked blood specimens. The results showed that the developed MIRA-LFD assay displayed a detection limit of 6 CFU/mL for detecting *A. baumannii* (**Figure 3A**), which was significantly better than that of real-time PCR method (**Figure 3B**).

### Evaluating spiked blood specimens for the MIRA-LFD assay

To determine the diagnostic validity of MIRA-LFD assay, 50 spiked blood specimens were used for the second step. From the **Table 2**, it can be seen that the newly MIRA-LFD assay had high specifcity and sensitivity for identifying *A. baumannii* in spiked blood specimens, which may become a powerful tool for rapid and reliable diagnosis of BSI caused by *A. baumannii* in primary hospitals.

**Table 2:** Characteristics of the Fifty isolates used for validation of the newly MIRA-LF assay.

No.	Bacterial strain	Location	Concentration (ng/µL)	MIRA-LF assay
1	*A. baumannii*	ICU	0.5	+
2	*A. baumannii*	ICU	0.5	+
3	*A. baumannii*	ICU	0.6	+
4	*A. baumannii*	ICU	0.7	+
5	*A. baumannii*	ICU	0.6	+
6	*A. baumannii*	ICU	0.8	+
7	*A. baumannii*	ICU	0.9	+
8	*A. baumannii*	ICU	0.8	+
9	*A. baumannii*	ICU	1.6	+
10	*A. baumannii*	ICU	1.2	+
11	*A. baumannii*	ICU	0.6	+
12	*A. baumannii*	GICU	0.8	+
13	*A. baumannii*	GICU	1.2	+
14	*A. baumannii*	GICU	1.8	+
15	*A. baumannii*	NICU	2.0	+
16	*A. baumannii*	NICU	0.6	+
17	*A. baumannii*	NICU	0.6	+
18	*A. baumannii*	NICU	0.9	+
19	*A. baumannii*	NICU	0.5	+
20	*A. baumannii*	CCU	0.6	+
21	*A. baumannii*	CCU	0.7	+
22	*A. baumannii*	CCU	0.6	+
23	*A. baumannii*	CCU	0.6	+
24	*A. baumannii*	CCU	0.8	+
25	*A. baumannii*	CCU	1.0	+
26	*A. baumannii*	RICU	0.6	+
27	*A. baumannii*	RICU	0.8	+
28	*A. baumannii*	RICU	0.6	+
29	*A. baumannii*	RICU	0.8	+
30	*A. baumannii*	RICU	1.6	+
31	*E. coli*	ICU	1.6	–
32	*E. coli*	ICU	0.9	–
33	*E. coli*	CCU	0.8	–
34	*E. coli*	CCU	0.6	–
35	*K. pneumoniae*	ICU	0.6	–
36	*K. pneumoniae*	ICU	1.6	–
37	*K. pneumoniae*	CCU	1.2	–
38	*K. pneumoniae*	CCU	0.8	–
39	*E. cloacae*	ICU	0.6	–
40	*E. cloacae*	ICU	0.8	–
41	*E. cloacae*	CCU	1.2	–
42	*E. cloacae*	CCU	1.5	–
43	*K. oxytoca*	CCU	0.6	–
44	*K. oxytoca*	ICU	0.8	–
45	*P. aeruginosa*	ICU	0.6	–
46	*P. aeruginosa*	ICU	0.9	–
47	*S. maltophilia*	ICU	1.6	–
48	*S. maltophilia*	ICU	1.2	–
49	*B. cepacia*	ICU	1.3	–
50	*B. cepacia*	ICU	0.8	–

+, positive.

-, negative.

## Discussion

Compared with other rapid molecular detection methods, MIRA-LFD assay has exhibited several advantages, such as high sensitivity, rapid detection time, convenient operation, and less requirement for specialized equipment. In addition, the MIRA amplification products can be detected by the naked eye with a lateral flow dipstick. Therefore, MIRA-LFD is superior to other rapid molecular detection methods. In this study, we report the development and validation of a MIRA-LFD assay for detection of *A. baumannii* in spiked blood specimens. The whole process from MIRA amplification to LFD detection takes about 15 min or less. The main results are as follows. First, the MIRA-LFD assay is more sensitive and specific than real-time PCR. Second, the MIRA-LFD assay is time-saving, easy to use, and has the lowest energy consumption. Third, the MIRA-LFD assay can meet the needs for rapid detection on first aid and emergency treatment, especially for resource-limited settings and poorly equipped laboratories.

In order to develop a set of experimental platforms capable of detecting *A. baumannii* for timely treatment of BSI, 16 pairs of primers and their corresponding probes were designed for *A. baumannii*. The best primer set was identified by forward and reverse screening experiments using agarose gel electrophoresis and used for subsequent MIRA-LFD experiments. To determine the optimum amplification temperature, we found that the MIRA-LFD assay worked well over a wide temperature range of 25-45°C, indicating that there was no significant impact on reaction performance over this temperature range. Furthermore, we also found that the positive results appeared after the amplification occurred for 5 min. In order to shorten the whole detection time and ensure the detection efficiency and sensitivity, the amplification time of MIRA-LFD was 10 min (Li et al., 2021).

The MIRA-LFD assay had a high species specificity that could detect all the *A. baumannii* isolates. In addition, there was no cross-reactivity with other non-*A. baumannii* bacteria species under the experimental conditions used, which indicates that MIRA-LFD has good specificity (Zhuo et al., 2022). Further studies should focus on verifying the potential cross-reactivity of DNA using the MIRA-LFD assay with other non-*A. baumannii* isolates. The bacterial DNA from spiked blood specimens was tested for MIRA-LFD assay’s sensitivity. Our results showed the sensitivity of established MIRA-LFD assay can up to 6 CFU/mL, which was better than that of real-time PCR. Next, we used the MIRA-LFD assay to detect clinical spiked blood specimens, and the results showed that 30 clinical *A. baumannii* isolates were retrospectively confirmed by the MIRA-LFD assay, and 20 clinical non-*A. baumannii* isolates showed negative results for *OXA51* gene detection. Therefore, the positive detection rate of the MIRA-LFD assay was 100%. There were no false positive results, indicating that the MIRA-LFD assay is very practical (Preston et al., 2016).

However, the limitation of this study is that the validation strains were limited in this study. To ensure the accuracy and reliability of the identification results, we need to further expand the number and types of validation strains to obtain better identification results (Li et al., 2019b; Vergara et al., 2020). Further studies are required to test the specificity and sensitivity of the present method on clinical blood culture samples that resulted positive for Gram negative bacteria at the Gram stain.

In summary, the MIRA-LFD assay is time-saving, more effective and sensitive than conventional identification methods, which has the potential to be applied in primary hospitals (Yin et al., 2017). Moreover, this method will become the mainstream of quantitative molecular detection of bacteria in the future (Schuler et al., 2015).

## Data availability statement

The original contributions presented in the study are included in the article/**Supplementary Material**. Further inquiries can be directed to the corresponding authors.

## Author contributions

W-WH, and JL completed data acquisitionn, data interpretation and drafted the manuscript. S-LG, and JL provided technical support and supervised the study. All authors contributed to the critical revision of the manuscript and approved the final version of the paper.

## Funding

This study was supported by Chongqing Natural Science Foundation (cstc2020jcyj-msxmX0207), Hospital Nurturing Fund of the First Affiliated Hospital of Chongqing Medical University (PYJJ2019-216), and the Graduate Scientific Research and Innovation Project of Chongqing (CYB21190).

## Acknowledgments

We appreciate the generosity of the Forhigh Biotechnology Corporation for providing the standard strain *A. baumannii* ATCC19606.

## Conflict of interest

The authors declare that the research was conducted in the absence of any commercial or financial relationships that could be construed as a potential conflict of interest.

## Publisher’s note

All claims expressed in this article are solely those of the authors and do not necessarily represent those of their affiliated organizations, or those of the publisher, the editors and the reviewers. Any product that may be evaluated in this article, or claim that may be made by its manufacturer, is not guaranteed or endorsed by the publisher.

## References

[B1] Abhari S. S. Azizi O. Modiri L. Aslani M. M. Assmar M. Fereshteh S.. (2021). Two new rapid pcr-based methods for identification of acinetobacter baumannii isolated from clinical samples. Mol. Cell. Probes 58, 101732. doi: 10.1016/J.Mcp.2021.101732 33878387

[B2] Chopra T. Marchaim D. Awali R. A. Krishna A. Johnson P. Tansek R.. (2013). Epidemiology of bloodstream infections caused by acinetobacter baumannii and impact of drug resistance to both carbapenems and ampicillin-sulbactam on clinical outcomes. Antimicrob. Agents Chemother. 57, 6270–6275. doi: 10.1128/Aac.01520-13 24100492 PMC3837851

[B3] Hamouda A. (2017). Identification of acinetobacter baumannii of human and animal origins by a gene-specific pcr. Curr. Microbiol. 74, 1118–1122. doi: 10.1007/S00284-017-1283-1 28664218

[B4] Hu F. Guo Y. Yang Y. Zheng Y. Wu S. Jiang X.. (2019). Resistance reported from China antimicrobial surveillance network (Chinet) in 2018. Eur. J. Clin. Microbiol. Infect. Dis. 38, 2275–2281. doi: 10.1007/S10096-019-03673-1 31478103

[B5] Kengkla K. Kongpakwattana K. Saokaew S. Apisarnthanarak A. Chaiyakunapruk N. (2018). Comparative efficacy and safety of treatment options for mdr and xdr acinetobacter baumannii infections: A systematic review and network meta-analysis. J. Antimicrob. Chemother. 73, 22–32. doi: 10.1093/Jac/Dkx368 29069421

[B6] Li J. Hu W. Zhang F. Li M. Rao C. Lu W. (2019b). Evaluation of matrix-assisted laser Desorption/Ionization time-Of-Flight mass spectrometry for identifying burkholderia pseudomallei and burkholderia thailandensis isolates. Eur. J. Clin. Microbiol. Infect. Dis. 38, 191–196. doi: 10.1007/S10096-018-3415-3 30426332

[B7] Li J. Macdonald J. (2015). Advances in isothermal amplification: Novel strategies inspired by biological processes. Biosens. Bioelectron. 64, 196–211. doi: 10.1016/J.Bios.2014.08.069 25218104

[B8] Liu J. Shu Y. Zhu F. Feng B. Zhang Z. Liu L.. (2021). Comparative efficacy and safety of combination therapy with high-dose sulbactam or colistin with additional antibacterial agents for multiple drug-resistant and extensively drug-resistant acinetobacter baumannii infections: A systematic review and network meta-analysis. J. Glob. Antimicrob. Resist. 24, 136–147. doi: 10.1016/J.Jgar.2020.08.021 32889142

[B9] Li T. T. Wang J. L. Zhang N. Z. Li W. H. Yan H. B. Li L.. (2019a). Rapid and visual detection of trichinella spp. using a lateral flow strip-based recombinase polymerase amplification (Lf-rpa) assay. Front. Cell Infect. Microbiol. 9. doi: 10.3389/Fcimb.2019.00001 PMC6348712 30719427

[B10] Li J. Zhong Q. Shang M. Y. Li M. Jiang Y. S. Zou J. J.. (2021). Preliminary evaluation of rapid visual identification of burkholderia pseudomallei using a newly developed lateral flow strip-based recombinase polymerase amplification (Lf-rpa) system. Front. Cell Infect. Microbiol. 11. doi: 10.3389/Fcimb.2021.804737 PMC8804217 35118011

[B11] Lobato I. M. O’sullivan C. K. (2018). Recombinase polymerase amplification: Basics, applications and recent advances. Trends Analyt. Chem. 98, 19–35. doi: 10.1016/J.Trac.2017.10.015 PMC7112910 32287544

[B12] Lu Y. Li M. C. Liu H. C. Lin S. Q. Zhao X. Q. Liu Z. G.. (2021). Detecting mycobacterium tuberculosis complex and rifampicin resistance via a new rapid multienzyme isothermal point mutation assay. Anal. Biochem. 630, 114341. doi: 10.1016/J.Ab.2021.114341 34411551

[B13] Ma W. Situ B. Lv W. Li B. Yin X. Vadgama P.. (2016). Electrochemical determination of micrornas based on isothermal strand-displacement polymerase reaction coupled with multienzyme functionalized magnetic micro-carriers. Biosens. Bioelectron. 80, 344–351. doi: 10.1016/J.Bios.2015.12.064 26855164

[B14] Perez F. Hujer A. M. Hujer K. M. Decker B. K. Rather P. N. Bonomo R. A. (2007). Global challenge of multidrug-resistant acinetobacter baumannii. Antimicrob. Agents Chemother. 51, 3471–3484. doi: 10.1128/Aac.01464-06 17646423 PMC2043292

[B15] Piepenburg O. Williams C. H. Stemple D. L. Armes N. A. (2006). Dna detection using recombination proteins. PloS Biol. 4, E204. doi: 10.1371/Journal.Pbio.0040204 16756388 PMC1475771

[B16] Preston S. Jabbar A. Nowell C. Joachim A. Ruttkowski B. Cardno T.. (2016). Practical and low cost whole-organism motility assay: A step-By-Step protocol. Mol. Cell. Probes 30, 13–17. doi: 10.1016/J.Mcp.2015.08.005 26365227

[B17] Schaad N. Schuenzel E. (2010). Sensitive molecular diagnostic assays to mitigate the risks of asymptomatic bacterial diseases of plants. Crit. Rev. Immunol. 30, 271–275. doi: 10.1615/Critrevimmunol.V30.I3.40 20370634

[B18] Schuler F. Schwemmer F. Trotter M. Wadle S. Zengerle R. Von Stetten F.. (2015). Centrifugal step emulsification applied for absolute quantification of nucleic acids by digital droplet rpa. Lab. Chip. 15, 2759–2766. doi: 10.1039/C5lc00291e 25947077

[B19] Shang M. Li J. Sun X. Su N. Li B. Jiang Y.. (2021). Duplex reverse transcription multienzyme isothermal rapid amplification assays for detecting SARS-CoV-2. Clin. Lab. 67, 2517–2524. doi: 10.7754/Clin.Lab.2021.210239 34758242

[B20] Sun M. L. Lai H. Y. Chong N. Y. Liu D. F. Zhang Z. Y. Pang B.. (2021). Simple and feasible detection of hepatitis b virus *via* combination of multienzyme isothermal rapid amplification and lateral flow dipstick strip. Front. Mol. Biosci. 8. doi: 10.3389/Fmolb.2021.763079 PMC8674754 34926579

[B21] Tada T. Uchida H. Hishinuma T. Watanabe S. Tohya M. Kuwahara-Arai K.. (2020). Molecular epidemiology of multidrug-resistant acinetobacter baumannii isolates from hospitals in Myanmar. J. Glob. Antimicrob. Resist. 22, 122–125. doi: 10.1016/J.Jgar.2020.02.011 32084608

[B22] Turton J. F. Woodford N. Glover J. Yarde S. Kaufmann M. E. Pitt T. L. (2006). Identification of acinetobacter baumannii by detection of the blaoxa-51-Like carbapenemase gene intrinsic to this species. J. Clin. Microbiol. 44, 2974–2976. doi: 10.1128/Jcm.01021-06 16891520 PMC1594603

[B23] Tu F. Zhang Y. Xu S. Yang X. Zhou L. Ge X.. (2022). Detection of pseudorabies virus with a real-time recombinase-aided amplification assay. Transbound Emerg. Dis. 69 (4), 2266–2274. doi: 10.1111/Tbed.14241 34273259

[B24] Vergara A. Boutal H. Ceccato A. López M. Cruells A. Bueno-Freire L.. (2020). Assessment of a loop-mediated isothermal amplification (Lamp) assay for the rapid detection of pathogenic bacteria from respiratory samples in patients with hospital-acquired pneumonia. Microorganisms 8 (1), 103. doi: 10.3390/Microorganisms8010103 PMC7022425 31940771

[B25] Xiong D. Dai W. Gong J. Li G. Liu N. Wu W.. (2020). Rapid detection of sars-Cov-2 with crispr-Cas12a. PloS Biol. 18, E3000978. doi: 10.1371/Journal.Pbio.3000978 33320883 PMC7737895

[B26] Yin F. Liu J. Liu A. Li Y. Luo J. Guan G.. (2017). Rapid diagnosis of theileria annulata by recombinase polymerase amplification combined with a lateral flow strip (Lf-rpa) in epidemic regions. Vet. Parasitol. 237, 125–129. doi: 10.1016/J.Vetpar.2017.02.019 28249769

[B27] Zarrilli R. Giannouli M. Tomasone F. Triassi M. Tsakris A. (2009). Carbapenem resistance in acinetobacter baumannii: The molecular epidemic features of an emerging problem in health care facilities. J. Infect. Dev. Ctries. 3, 335–341. doi: 10.3855/Jidc.240 19759502

[B28] Zhuo X. Zhao J. Wang L. Sun B. Sun L. Wang C.. (2022). Development and evaluation of a multiplex quantitative polymerase chain reaction assay for detecting bacteria associated with lower respiratory tract infection. Int. J. Infect. Dis. 122, 202–211. doi: 10.1016/J.Ijid.2022.05.052 35644352

