# Supplementary material for: Development and evaluation of a rapid and sensitive multienzyme isothermal rapid amplification with a lateral flow dipstick assay for detection of Acinetobacter baumannii in spiked blood specimens
Source: Front Cell Infect Microbiol. 2022 Oct 19;12:1010201. doi: 10.3389/fcimb.2022.1010201 (PMC9626983; doi:10.3389/fcimb.2022.1010201)
Supplement: Supplementary file 1 [file DataSheet_1.docx]

**Title:** Development and evaluation of a rapid and sensitive multienzyme isothermal rapid amplification with a lateral flow dipstick assay for detection of *Acinetobacter baumannii* in spiked blood specimens

**Authors:** Wei-Wei Hu^1^, Jian-Wei He^2^, Shu-Liang Guo^1*^, Jin Li^2^^*^

***Co-corresponding authors:**

Jin Li, M.D. Ph.D., Email: jamly1110@163.com

Shu-Liang Guo, M.D. Ph.D., Email: guosl999@sina.com

^1^Department of Respiratory and Critical Care Medicine, The First Affiliated Hospital of Chongqing Medical University, Chongqing, 400016, P.R.China

^2^Key Laboratory of Clinical Laboratory Diagnostics (Ministry of Education), College of Laboratory Medicine, Chongqing Medical University, Chongqing, 400016, P.R. China

Tel: +86-23-89012745

Fax: +86-23-89012745

**SUPPLEMENTARY MATERIALS INDEX**

**Supplementary Figure 1. The schematic workflow of the developed MIRA-LF assay for detecting *A. baumannii*.**

**Supplementary Figure 2. Primer screening for basic MIRA assays.**


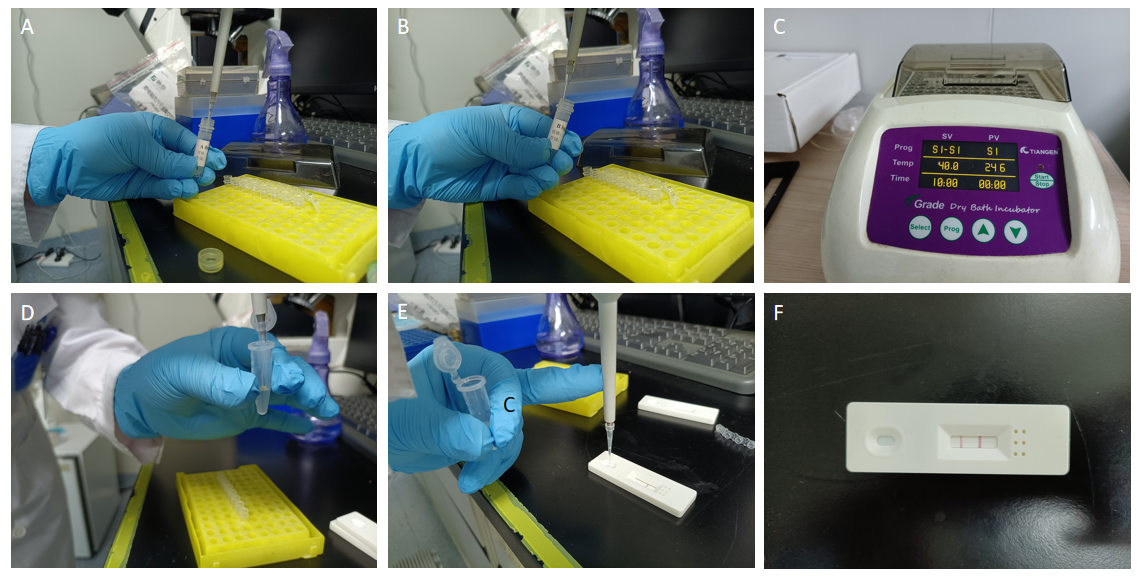


**Supplementary Figure 1. The schematic workflow of the developed MIRA-LFD assay for detecting *A. baumannii*.** The operation steps were as follows: **A**, 29.4 μL A buffer, 12.1 μL double-distilled water, 2 μL forward primer (10 μM), 2 μL reverse primer (10 μM), 2 μL sample were added in the reaction tube. **B**, 2.5 µL B buffer (280 mM) were added in the same reaction tube, and then the tubes were closed carefully, vortexed and centrifuged briefly. **C**, The above mixed reagents were immediately placed in a matched metal heat block and incubated at 40℃ for 10 minutes. **D**, The amplicons of MIRA were diluted 20-fold in buffer. **E**, 50 µL of amplification dilution was added to the colloidal gold reaction tank. **F**, Visual detection of the amplicons of MIRA with the help of lateral flow strips.


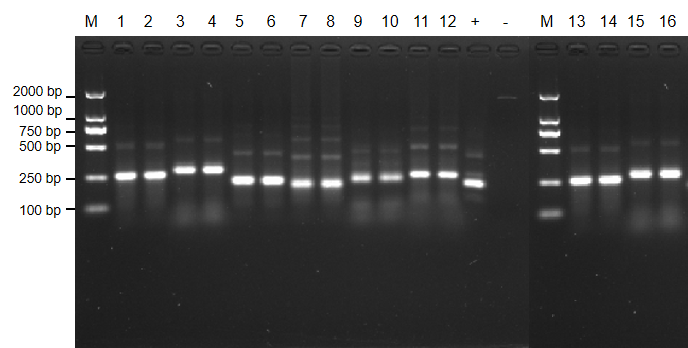


**Supplementary Figure 2. Primer screening for basic MIRA assays.** A total of 16 pairs of specific primers targeting the *OXA51* gene of *A. baumannii* were designed for screening by basic MIRA (2 ng gDNA as template, at 40°C for 20 min). Lanes 1-16 are the results of electrophoresis of primers F1/R1, F1/R2, F1/R3, F1/R4, F2/R1, F2/R2, F2/R3, F2/R4, F3/R1, F3/R2, F3/R3, F3/R4, F4/R1, F4/R2, F4/R3, F4/R4. M: DNA marker.
